# Dataset of an actual life-risk insurance portfolio

**DOI:** 10.1016/j.dib.2022.108655

**Published:** 2022-10-03

**Authors:** Josep Lledó, Jose M. Pavía

**Affiliations:** a Department of Applied Economics, Universitat de Valencia, Spain; b Department of Applied Economics, GIPEyOP, Universitat de Valencia, Spain

**Keywords:** Life-risk insurance, Quarterly life tables, Capital at risk, Pricing, Actuarial

## Abstract

The foundation of the insurance business is built on data, the latter being one of the most valuable assets of any insurer. In fact, the risk structure to which an insurance company is exposed can actually be deduced by reviewing its customer database. It is not surprising, therefore, that access to real insurance datasets is very limited. This paper introduces and describes a dataset corresponding to a cross-section extraction of a real life-risk insurance portfolio. The dataset contains information on 76,102 policies and a total of 15 variables, including the capital at risk, the genders and dates of birth of the insured, and the effective and renewal dates of their policies. This dataset can be used both in teaching and in research. Combined with external life tables, the data available in the dataset can be used to build and compare pricing systems, to evaluate marketing strategies, in portfolio analysis, for calculations required by Solvency II regulations or for market benchmarking analysis. For example, the data from this dataset have been used in Pavía and Lledó [1] to compare the classic pricing methodology based on annual life tables with a new pricing methodology based on life tables with less than annual periodicity Pavía and Lledó [2], specifically quarterly, and in Lledó et al. to demonstrate the impact that using a new methodology to manage catastrophic risks in life insurance would have in terms of solvency capital requirements.

**Specifications Table**

**Table:** 

Subject	Business, Management and Decision Sciences; Economics, Econometrics and Finance
Specific subject area	Insurance; Finance and Banking; Strategy and Management; Safety, Risk, Reliability and Quality; Microeconomics
Type of data	Table (spreadsheet)
How the data were acquired	The main variables of the dataset have been obtained from a life insurance company that markets its products through the bancassurance channel. Secondary variables have been calculated directly by the authors on the basis of the principal variables.
Data format	Raw, analysed and filtered
Description of data collection	The data were obtained from the underwriting department of an insurance company through a monthly statement. The data are entered individually by clerks with insurance backgrounds. Prior to data storage, several checks evaluate both typographical errors and underwriting standards.
Data source location	The data have been obtained from an insurance Spanish company. Data anonymised.
Data accessibility	A data file (spreadsheet) is supplied as supplementary material with this article and is also available in OPENICPSR: https://doi.org/10.3886/E178881V1

## Value of the Data

• This dataset enables the analysis and study of the composition of a real life-risk insurance portfolio through its main variables (age, time of subscription, capital, gender, etc.).
• Combined with external life tables, this dataset contains the data with the minimum technical characteristics necessary to calculate risk-life insurance premiums, using both an annual and a sub-annual calculation basis.
• Actuarial students, trainers and researchers can benefit from these data to simulate pricing and reserving practices and to test new ideas and methodologies.
• Analysis of the data allows multiple economic indicators (KPIs) typical of the insurance sector to be collated, such as the Best Estimate Liabilities or the Solvency Capital Requirement in the Solvency II regulatory environment, or the life insurance loss ratio related to accounting management.
• This dataset can be used to carry out commercial actions on a certain group and/or as a market benchmark for an insurance company.

## 1 Data Description

This paper describes the anonymised data related to the policies in force on December 31, 2009 in a portfolio of life-risk insurance of a Spanish insurance company. The data sheet of the spreadsheet that accompanies this document (also available in https://doi.org/10.3886/E178881V1) consists of 76,102 rows and 15 columns, with each row corresponding to a policy and each column to a variable. Table 1 shows a brief description of the 15 variables available in the dataset. The set of variables can be divided into 5 main variables, extracted directly from the information available in the company's database, and 10 secondary variables, derived from the main variables through different calculation processes that are detailed in the next section. The 15 variables can be grouped into five categories according to their nature: (i) binary (*Gender*), (ii) ordinal (*ID, Month, r, s*), (iii) discrete-type quantitative data (*Age_Actuarial, Age_actuarial_quarter*), (vi) continuous-type quantitative data (*Capital, Age, t, x*) and (*v*) dates (*Birth_Date, Effective_Date, Renewal_Date and Birthday*).

**Table 1:** Variables in the dataset.

Variable	Description
ID	Unique identification code of the policy/insured (an integer number).
Gender	Gender of the insured (M = male, F = female).
Birth_Date	Date of birth of the insured (DD/MM/YYYY).
Effective_Date	Effective start date of the contractual relationship between the insurance company and the insured (DD/MM/YYYY).
Capital	Capital at risk, amount of money to be received by beneficiaries when the insured person dies (in €).
Renewal_Date	Date of renewal during the year of the insurance contract (DD/MM/YYYY).
Age	Exact age of the insured, measured in years, at the renewal date (a real number). The time elapsed between the date of birth and the renewal date.
t	Fractional age in years (0≤t<1) of the insured at the moment of the *Renewal_Date*. Number of years elapsed between the date of his/her last birthday and the renewal date (a real number between 0 and 1).
Age_Actuarial	Age of the insured, measured in whole years, obtained by rounding to the closest integer the exact (decimal) age of the insured (an integer number).
Birthday	Birthday date in 2009 (DD/MM/YYYY).
x	Time elapsed in years (0≤x<1) between the start of the year (0:00AM on 1 January) and the moment of the *Renewal_Date* (a real number between 0 and 1).
r	Ageing quarter (1 = 1Q, 2 = 2Q, 3 = 3Q, 4 = 4Q). Age-quarter of the insured at the time of renewal.
s	Seasonal quarter (1 = Winter, 2 = Spring, 3 = Summer and 4 = Autumn). Season-quarter of the moment of renewal. Please see details in the text.
Age_actuarial_quarter	Age of the insured, measured in years, after approximating his/her exact age to the closest integer age-quarter (a real number).
Month	Month of renewal date (1 = January, 2 = February, 3 = March, 4 = April, 5 = May, 6 = June, 7 = July, 8 = August, 9 = September, 10 = October, 11 = November, 12 = December) .

**Fig. 1:**
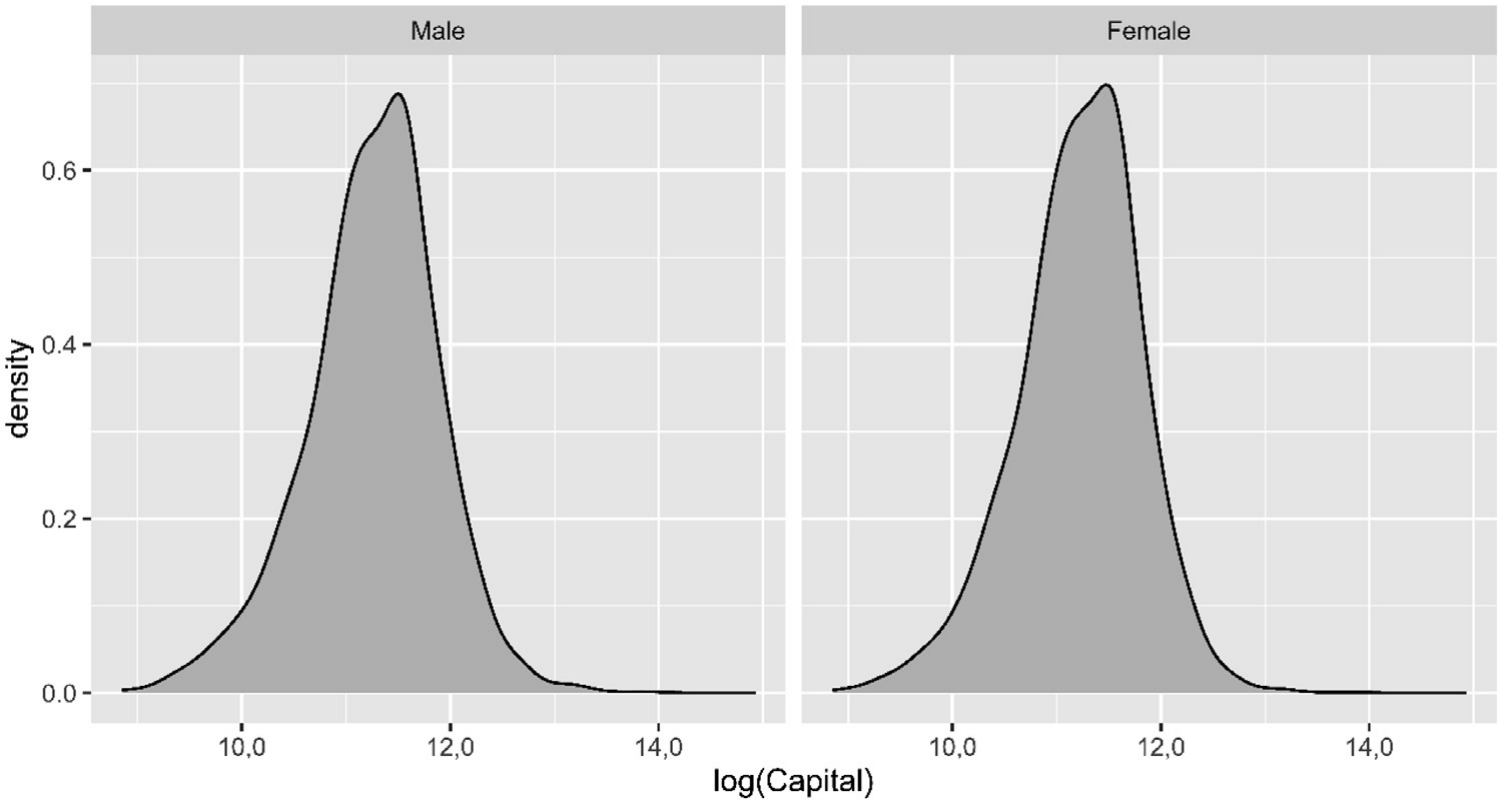
Empirical kernel density estimates of the distributions of capital at risks (*Capital* variable), in the log-scale, by gender obtained using 0.15 as bandwidth.

The *ID* variable is a numeric identifier of the insured (policy) and is unique for each row. The *Gender* variable represents the sex of the insured, with “M” and “F” denoting male and female, respectively. The *Birth_Date* variable corresponds to the date of birth of the insured. The format of all dates in the dataset is DD/MM/YYYY, where DD, MM, and YYYY represent the day of the month, month of the year, and year, respectively. The *Effective_Date* variable represents the start date of the contractual relationship between the insured and the insurance company regarding the life-risk insurance policy. This is the date on which the policy came into effect and covers the contingency of death while the policy is renewed. The variable *Capital* refers to the economic amount that the beneficiaries would receive in the event of the occurrence of the contracted coverage, in this case death. This variable is expressed in euros and the minimum contracting capital is €7000, while the maximum, corresponding to ID 23259, is a capital of €3,010,000. A graphical representation of the distribution of this variable by gender is displayed in Fig. 1, which shows in the log-scale the empirical kernel density estimates (continuous histogram) of the distributions of capital at risks obtained using the function density of the statistical software R, version 4.1.0 [4].

In addition to the above 5 variables, the dataset also contains 10 secondary (derivate) variables, which can be clustered into two groups depending on either their statistical characteristics or their usefulness. In terms of characteristics, on one side are the variables related to the dates of age, contract date, renewal or evaluation date and on the other side are those variables derived from the representation of the event on the double time scale (age, calendar) typical of the Lexis scheme (see e.g., [5]), related to the time of occurrence of the event within the reference year. In terms of utility, the variables can be classified into two blocks; those to be used with annual life tables and those that are also necessary to manage subannual risks.

**Fig. 2:**
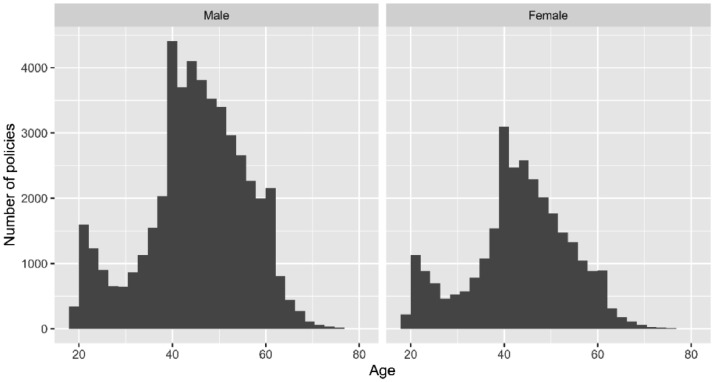
Number of policies in the portfolio by age for men (left panel) and women (right panel).

The *Renewal_Date* and *Birthday* variables represent, respectively, the times of renewal and birthday of the policy in the valuation year. The *Age* variable measures the exact age of the insured at the time of renewal and the *Age_Actuarial* variable the actuarial age at the time of renewal. Fig. 2 shows graphically, through histograms, the number of policies by gender in the portfolio for each age. As can be seen, this dataset has significantly more policies covering the risk of dying for men than for women. Of the total insured, 47,652 (62.62%) are men while 28,450 (37.38%) are women. The age distribution of the number of policyholders (see Fig. 2) shows a young portfolio, where the majority of policyholders fall between the ages of 40 and 60 and a peak in the age range between 21 and 24 years. The distribution is similar for both sexes.

The last block of variables available in the dataset are those needed to deal with quarterly (sub-annual) life tables, for which demographic events need to be located using a system of coordinates in each 1×1-year surface of the Lexis scheme. Within each annual area of the Lexis scheme, each renewal event is placed using two coordinates (*x, t*), where the variable/coordinate *x* captures the value of the Lexis coordinate corresponding to the calendar axis in the year of renewal (i.e., the time elapsed in years between the start of the year and the moment of the *Renewal_Date*) and the variable/coordinate *t* the decimal value of the Lexis coordinate corresponding to the age axis in the year of renewal (i.e., the time elapsed in years between the date of last birthday of the insured and the renewal date). The variable *x*, together with *t*, is used to derive the variables *r* and *s* [2]. The variables *r* and *s*, which take values in the set {1, 2, 3, 4}, allow each policy to be placed in the quarter of age and calendar where the event occurs (in this case the renewal, taking into account the exact age of the insured). Each policy is attached a value {1, 2, 3, 4} for *s* and *r* based on the quarter-length subinterval of the [0, 1] interval in which *x* and *t* lie on.

**Fig. 3:**
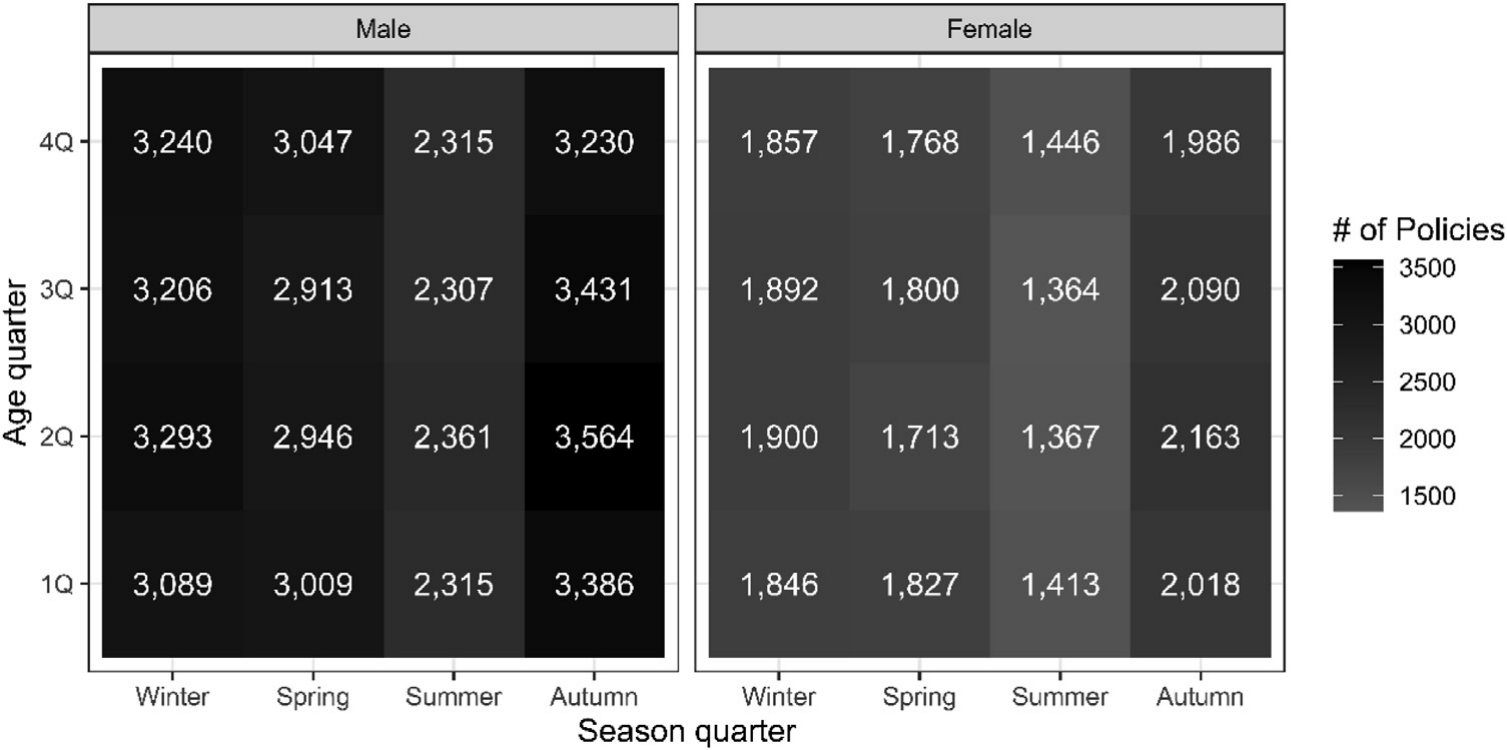
Number of policies in the portfolio that renew in each combination of quarter of age and calendar, (r,s)-quarter, by gender. The x-axis refers to the calendar quarter in Spain: winter (January, February and March), spring (April, May and June), summer (July, August and September) and autumn (October, November and December). The correspondences between months and seasons are approximate.

Given the significant impact that the age-season interaction has as people get older on the risk of dying and the fact that this is higher during winter [2], [3], it is usually worthwhile to identify calendar quarters using seasons. In this case, as the dataset is from Spain (a country located on the North hemispheric), the values (1, 2, 3 and 4) for *s* correspond to winter (January, February and March), spring (April, May and June), summer (July, August and September) and autumn (October, November and December), respectively. Note that the correspondences between months and seasons are approximate. Fig. 3 shows (aggregated by ages) the number of policies in the portfolio that, by gender, renew for each combination of aging- and seasonal-quarter. Finally, the *Age_actuarial_quarter* variable represents the quarterly actuarial age of the insured at the time of renewal and *Month* the month of renewal.

Table 2 summarises the main statistics of the quantitative variables. For the *Capital* variable, the mean is higher than the median, a consequence of high outliers (see Fig. 1). The variables related to age (*Age, Age_Actuarial* and *Age_Actuarial_quarter*) show, in line with that seen in Fig. 2, a concentration of insured between 39 (first quartile) and 52 years old (third quartile), with the value of the mean and median being similar. The numerical result of the variable *t (x)* shows that the average time between the renewal date and the date of the last birthday (beginning of the year) is half a year.

**Table 2:** Summary of the quantitative variables in the dataset.

	Capital	Age	Age_Actuarial	Age_Actuarial_quarter	t	x
Min	7000 €	17.89	18	18	0	0
1st Quartile	54,000 €	38.59	39	38	0.248	0.241
Median	80,000 €	45.14	45	45	0.494	0.493
Mean	91,452 €	44.48	44.48	44.11	0.498	0.509
3rd Quartile	110,000 €	51.96	52	52	0.750	0.797
Max	3,010,000 €	78.84	79	78	0.999	0.997

## 2 Experimental Design, Materials and Methods

The accessibility of public data on insured populations is limited and only available for certain lines of business. For non-life insurance, there are databases related to car insurance (e.g., [6,7]), property (e.g., [8]) and health (e.g., [9]). However, with regard to life insurance, the information available on real portfolios is particularly scarce and the data available are usually aggregated, such as in the case of the data found in Forfar et al. [10]. However, for a payment of relatively high annual fees, it is possible to access more detailed data. For example, organisations such as the Continuous Mortality Investigation Bureau have accumulated and analysed data on mortality and morbidity risks arising under life assurance, annuity and pension business for nearly 90 years.

In life-risk insurance, the insured, through the payment of a premium, guarantee that in the event of death the beneficiaries of the policy receive a monetary capital. Insurance companies are the entities that offer these products to the market. The dataset used in this paper corresponds to the basic information available in the computer files of an insurance company that operates in the life-risk business in Spain in a particular moment in time. Each row in the spreadsheet corresponds to a year-term life insurance policy, a product that is automatically renewed every year. The insurance ceases to be in force when either the insured decides to cancel the policy or s/he reaches a certain advanced age. The data extraction date (i.e., the valuation date) of the dataset corresponds to 24:00 on 31/12/2009 (quarterly and annual closing). As this is a point in time, the dataset, implicitly, already reflects inflows (new business) and outflows (withdraws and deaths) of the insurance portfolio.

The five main variables (*ID, Gender, Birth_Date, Effective_Date* and *Capital*) of the dataset are obtained directly from the company's database and make up part of the features contained in the policies in accordance with their underwriting policy. Personal identifiers were replaced by ordered numerical values (*ID* variable) to safeguard the confidentiality of all policyholders. The underwriting policy allows people with an entry age of between 18 and 79 years of actuarial age (see variable *Age_Actuarial*) to take out a policy, meaning the variable *Birth_Date* is less than 31/12/1997 and greater than 01/01/1930.

The risk capital of each policy (*Capital* variable) is set by the policyholder at the time the policy is issued, *Effective_Date*, and can be modified, at the request of the insured, at each of the renewal dates, *Renewal_Date*. The company has a minimum subscription capital of €7000. The capital is usually several times the annual salary of the insured and its main objective is to compensate the beneficiaries financially in the event of the insured´s death. The most common capital chosen by policyholders (see Table 2) lies between €54,000 (first quartile) and €110,000 (third quartile), but may exceed more than two million euros for a few policyholders. In reality, most of the capitals are between the first two values, and in this portfolio the capitals contracted by men are slightly higher than the values for women.

Apart from the main variables, other variables can be derived which are needed for carrying out, for example, the pricing and reserving processes. The variable *Age*, which measures the time in years between the dates of renewal (*Renewal_Date*) and birth (*Birth_Date*), is calculated as the time distance in years between both variables, taking into account the average number of days in a year, 365.25 days. The variable *t* is calculated from this as the fractional part in years (0≤t<1).

The use of an annual life table during the pricing and reserving processes requires the use of an integer age at the time the policy comes into effect, *Effective_Date*, and renewal, *Renewal_Date*. The usual practice in the insurance industry is to round the *Age* variable to the nearest integer, which gives rise to the *Age_Actuarial* variable.

The typical calculations in life insurance are made using an annual life table. This table summarises the mortality experience of a group that presents certain homogeneous characteristics. However, recent research carried out by Pavía and Lledó [1] highlights the need to use less than annual periodicities to perform pricing calculations, given that mortality patterns are not uniform throughout the year [2]. This dataset has the necessary variables that allow life-risk insurance products to be priced on an annual and quarterly basis.

The variable *x* measures the time elapsed in years (0≤x<1) between the start of the year (0:00AM on 1 January) and the moment of the *Renewal_Date*, within the year. The number of years between the *Renewal_date* and the previous year valuation date at 24:00 is calculated by dividing this by the number of days, 365, except in a leap year when it is 366. Using quarterly life tables requires the use of two new variables, which can be derived from *x* and *t*. The variable *r* coincides with the nearest ageing-quarter of the variable *t* and the variable *s* with the nearest seasonal-quarter of the variable *x*. The variables *r* and *s* take values in the set {1, 2, 3, 4}, from the equations r=⌊4t⌋+1 and s=⌊4x⌋+1, where ⌊·⌋ is the floor function (i.e., the function that, for any real number, computes the greatest integer number less than or equal to it). Finally, the quarterly actuarial age, *Age_actuarial_quarter*, refers to the actuarial age that the insured would have at the time of renewal on a scale of quarters, and is calculated (in years) by approximating the exact age to the nearest quarter age, that is, equal to ⌊Age⌋+[4x]/4, where [·] stands for the rounding function.

The distribution of the variable enables identification of the effective quarter in which the policy is renewed or, alternatively, the quarter in which the insured took out the policy for the first time. If promotion of the product, led by the marketing department, is uniform throughout the year, the values of *s* would be expected to be similar for each of the four calendar quarters. As can be seen in Fig. 3, in this portfolio the sale of policies per season is not homogeneous throughout the year. Although the number of policies sold in the first two semesters is quite similar (the second quarter presents slightly lower values), a significant decrease is observed in the third quarter, coinciding with the summer holiday period (mainly July and August) in Spain. Sales grow again in the last quarter, where companies are more aggressive as they work to balance budgets and meet targets. At the beginning of the year, insurance companies set their sales targets for the entire year, but it is not until the last few months of the year that the commercial network instigates their main commercial campaigns aimed at selling insurance products. With regard to the distribution of the variable *r*, the age-quarter, this is seen to be distributed fairly uniformly over the four quarters.

## Ethics Statements

The relevant informed consent was obtained by the company from the insured in the moment of contracting the product. Data is offered anonymised.

## CRediT authorship contribution statement

**Josep Lledó:** Methodology, Software, Visualization, Resources, Data curation, Investigation, Writing – original draft, Writing – review & editing. **Jose M. Pavía:** Conceptualization, Methodology, Supervision, Visualization, Funding acquisition, Investigation, Writing – review & editing.

## Declaration of Competing Interest

The authors declare to have no known competing financial interests or personal relationships that may have influenced the work reported in this paper.

## Data Availability

Portfolio (Original data) (ICPSR).

## Appendix Supplementary materials

Image, application 1
